# Boundaries in metagenomic screenings using *lac*Zα-based vectors

**DOI:** 10.1590/1678-4685-GMB-2018-0252

**Published:** 2020-03-06

**Authors:** Luana de Fátima Alves, Tiago Cabral Borelli, Cauã Antunes Westmann, Rafael Silva-Rocha, María-Eugenia Guazzaroni

**Affiliations:** 1 Universidade de São Paulo, Faculdade de Filosofia, Ciências e Letras de Ribeirão Preto, Departamento de Biologia, Ribeirão Preto, SP, Brazil.; 2 Universidade de São Paulo, Faculdade de Medicina de Ribeirão Preto, Departamento de Bioquímica e Imunologia, Ribeirão Preto, SP, Brazil.; 3 Universidade de São Paulo, Faculdade de Medicina de Ribeirão Preto, Departamento de Biologia Celular e Molecular, Ribeirão Preto, SP, Brazil.

**Keywords:** Functional metagenomics, protease, glycosyl hydrolase, false positive clones

## Abstract

Metagenomics approaches have been of high relevance for providing enzymes used in diverse industrial applications. In the current study, we have focused on the prospection of protease and glycosyl hydrolase activities from a soil sample by using the *lacZ*α *-*based plasmid pSEVA232. For this, we used a functional screen based on skimmed milk agar and a pH indicator dye for detection of both enzymes, as previously reported in literature. Although we effectively identified positive clones in the screenings, subsequent experiments revealed that this phenotype was not because of the hydrolytic activity encoded in the metagenomic fragments, but rather due to the insertion of small metagenomic DNA fragments *in frame* within the coding region of the *lac*Z gene present in the original vector. Analyses of the thermodynamic stability of mRNA secondary structures indicated that recovering of positive clones was probably due to higher expression levels of the chimeric lacZα-genes in respect to the original from empty vector. We concluded that this method has a higher tendency for recovery false positive clones, when used in combination with a *lacZ*α*-*based vector. As these vectors are massively used in functional metagenomic screenings, we highlight the importance of reporting boundaries in established metagenomic screenings methodologies.

## Introduction

Renewable resources, such as plant biomass (essentially lignocellulose), have a significant potential for the production of biofuels and other biotech-produced industrial chemicals due to their higher abundancy and lower price in comparison to other commercial substrates (Simmons *et al.*, 2010). However, the physicochemical constraints placed on cellulose and hemicellulose polymers by lignin made the saccharification procedure an expensive process due to a lack of biocatalysts tolerant to process-specific parameters (Klein-Marcuschamer *et al.*, 2012; Papoutsakis, 2015). The notorious resilience of bacteria against environmental fluctuations and its inherent biochemical diversity allows screening and isolation of novel enzymes that are essential for effectively overcoming these barriers. Thus, there is a huge amount of gene resources held within the genomes of uncultured microorganisms, and metagenomics is one of the key technologies used to access and explore this potential (Dinsdale *et al.*, 2008; Fernández-Arrojo *et al.*, 2010; Mair *et al.*, 2017).

Functional metagenomics aims to recover genes encoding proteins with a valuable biochemical function (Lorenz and Eck, 2005; Fernández-Arrojo *et al.*, 2010; Mair *et al.*, 2017). For instance, genes considered of interest are the ones encoding: enzymes; adaptive proteins, conferring resistance to diverse physical or chemical stressors; catabolic pathways or even biosynthetic clusters involved in the production of bioactive compounds (Alves *et al.*, 2017). The functional metagenomic approach presents two different strategies for libraries generation. Primarily, large-insert libraries, constructed in cosmids or fosmids, allow for the stable recovery of large DNA fragments and sequence homology screening purposes (Danhorn *et al.*, 2012). This strategy would also allow the recovery of complete biosynthetic pathways or the functional expression of large multi-enzyme assemblies (as in the case of polyketide synthases or hydrogenases clusters) (Guazzaroni *et al.*, 2010, 2015). On the other hand, small-insert expression libraries (i.e., lambda phage vectors and plasmids), are constructed for activity screening from single genes or small operons (Danhorn *et al.*, 2012). In this strategy, strong vector expression signals (e.g., promoter and ribosome binding site) are used to guarantee that small DNA fragments (2-10 kb) cloned in the vector reach a good chance of being expressed and detected by activity screens (Ferrer *et al.*, 2008; Guazzaroni *et al.*, 2015). At this point, it is of particular relevance mentioning that *lacZ*α*-*based vectors are frequently used in different screenings, with high prevalence in small-insert expression metagenomic libraries (Lämmle *et al.*, 2007; Mirete *et al.*, 2007; Guazzaroni *et al.*, 2013; Morgante *et al.*, 2015; Gao *et al.*, 2016; Zhou *et al.*, 2016). In this sense, the blue/white screening, inherent of α-based vectors is one of the most common molecular techniques that allows detecting the successful ligation, and subsequently expressing the gene of interest in a vector (Zamenhof and Villarejo, 1972; Langley *et al.*, 1975; Ausubel *et al.*, 2003). 

Metagenomics strategies have been of high relevance for providing enzymes used in manufacturing applications (Schloss and Handelsman, 2003; Lorenz and Eck, 2005; Fernández-Arrojo *et al.*, 2010). The use of enzymes in industry has grown considerably, and a number of different categories of enzymes has been used in a wide variety of applications (Schoemaker, 2003). For example, proteases have been used in detergents, in pharmaceutical and chemical synthesis industries to degrade proteins into amino acids (Gupta *et al.*, 2002). Glycosyl hydrolases, which catalyze the hydrolysis of carbohydrates to sugars, have been applied to many processes further than bioethanol production (i.e., cellulose and hemicellulose conversion to fermentable sugars), being highly relevant in the textile, paper and food production industries (Kirk *et al.*, 2002).

**Figure 1:**
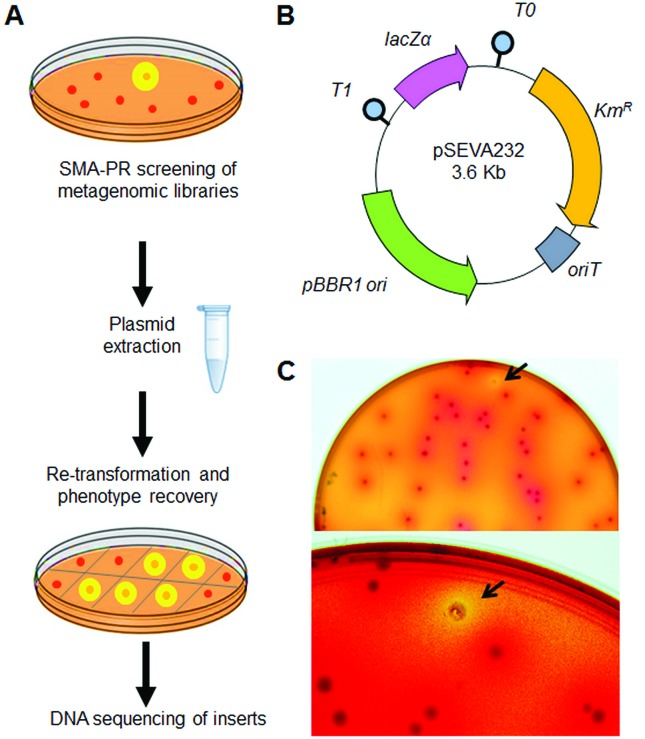
Schematic representation of the workflow for finding novel enzymes (proteases and GHs) using skimmed milk agar (SMA) and phenol red as pH indicator. (A) Schematic workflow showing functional metagenomic screening, selection of positive clones, checking of phenotype maintenance and sequencing of the metagenomics inserts. (B) Overall organization of the structure of pSEVA232 plasmid. Plasmid backbone includes antibiotic resistance marker (KmR), conjugation origin (oriT), broad host-range origin of replication (pBBR1), T1 and T0 transcriptional terminators and lacZa reporter, which contains a multiple cloning site (MCS) where metagenomic fragments were placed. (C) Plate of SMA-PR media after incubation at 37 ºC for 24 h. Arrow indicates a colony of E. coli surrounded by a yellow halo and identified as positive clone.

Studies found in the literature have reported that both enzymatic activities (protease and glycosyl hydrolase) could be found in a single pH-based assay using skimmed milk agar (SMA) (Jones *et al.*, 2007; Popovic *et al.*, 2015). These authors stated that the use of pH indicator dyes such as phenol red or bromophenol blue increases the sensitivity of the assay allowing detection of the acidic shift during hydrolysis of lactose by glycosyl hydrolases (detected as a yellow halo), or casein by proteases (visualized as clear halos) (Jones *et al.*, 2007; Popovic *et al.*, 2015). Hence, subsequent experiments should be done to identify the specific enzymatic activity of the recovered clones (Jones *et al.*, 2007). Therefore, in the current study we were interested in obtaining protease and glycosyl hydrolase activities from the microbial communities inhabiting a soil sample of a Secondary Atlantic Rain Forest (L. de F. Alves, unpublished results). For this, we implemented a metagenomic approach using a functional screen based on SMA and a pH indicator dye (Figure 1A). The metagenomic library was constructed in *Escherichia coli* as a host using the broad host-range vector pSEVA232, which is a *lacZ*α*-*based plasmid (Silva-Rocha *et al.*, 2013) (Figure 1B).

By implementing the SMA-phenol red (SMA-PR) screening approach, we effectively obtained nine clones that were able to generate the typical yellow halos indicative of glycosyl hydrolase (GH) production - although no clear halos, indicative of protease activity, were obtained. However, subsequent experiments revealed that the phenotype observed in these clones was not caused by exogenous genes providing hydrolytic activity. Unexpectedly, restriction profile analyses and sequencing of metagenomic inserts showed that the metagenomic fragments were too small for encoding enzymes able to display activity, even though the library was constructed using fragments of 2-7 kb and presented an average insert size of 4.08 kb. Further analyses showed that the metagenomic DNA fragments were inserted *in frame* with the coding region of the *lac*Z gene present in the original vector (α peptide of the β-galactosidase; Table S1). We concluded that the current SMA-PR method to obtain proteases and GHs has a higher tendency for false positive clones’ recovery, when used in combination with a *lacZ*α*-*based vector. As these vectors are massively used in screenings of small-insert expression libraries (Lämmle *et al.*, 2007; Mirete *et al.*, 2007; Guazzaroni *et al.*, 2013; Morgante *et al.*, 2015; Gao *et al.*, 2016; Zhou *et al.*, 2016), a robust strategy and previous experimental planning should be done to avoid finding and characterizing false positives clones.

## Materials and Methods

### Bacterial strains, plasmids and general growth conditions

*E. coli* DH10B (Invitrogen) cells were used for cloning, metagenomic library construction, and experimental procedures. *E. coli* cells were routinely grown at 37 ºC in Luria-Broth medium (Ausubel *et al.*, 2003). When required, kanamycin (50 μg/mL) was added to the medium to ensure plasmid retention. Transformed bacteria were recovered on LB liquid medium for 1 h at 37 °C and 180 rpm, followed by plating on LB-agar plates at 37 °C for at least 18 hours. Plasmids used in the present study were pSEVA232, pSEVA242 (Silva-Rocha *et al.*, 2013) and pSEVA242 bearing a 1.5 Kb insert (pSEVA242-1.5 kb) (this study), corresponding the endoglucanase *cel5*A gene from *Bacillus subtilis* 168 (Santos *et al.*, 2012).

### Nucleic acid techniques

DNA preparation, digestion with restriction enzymes, analysis by agarose gel electrophoresis, isolation of DNA fragments, ligations, and transformations were done by standard procedures (Ausubel *et al.*, 2003). Plasmid DNA was sequenced on both strands using the ABI PRISM Dye Terminator Cycle Sequencing Ready Reaction kit (PerkinElmer) and an ABI PRISM 377 sequencer (Perkin-Elmer) according to the manufacturer’s instructions.

### Screening of GH and protease activities

The metagenomic library used in this study (named LFA-USP3) was previously generated (L. de F. Alves, unpublished results) from a Secondary Atlantic Forest soil sample collected at the University of Sao Paulo, Ribeirão Preto, Brazil (21º0958.4S, 47º5120.1W). The library was constructed from a microbial community of a soil bearing specific tree litter composition (*Phytolacca dioica*). Metagenomic DNA was cloned into the pSEVA232 vector, a plasmid able to replicate in different gram-negative bacteria, due to its broad-host origin of replication (Silva-Rocha *et al.*, 2013). Briefly, soil metagenomic DNA was extracted using the UltraClean Soil DNA isolation kit (Mo Bio, EUA), partially digested using *Sau*3AI, before the fragments of 2-7 kb were selected and cloned into a *Bam*HI-digested pSEVA232 vector. *E. coli* DH10B cells were transformed with the resultant plasmids and the library presented about 257 Mb of eDNA distributed into approximately 63,000 clones harboring insert fragments with an average size of 4.08 kb.

Screening of GH and protease activities was performed according to Jones *et al.* (2007). The library clones were grown in LB-agar plates containing 1% (w/v) skimmed milk, 0.25 mg/mL phenol red and kanamycin (50 μg/mL) for 24 h at 37 °C. Colonies surrounded by a yellow halo against a red background were taken as potential positive clones, and plasmids were extracted for re-transformation in *E. coli*. Lastly, clones that maintained the phenotype were selected and their plasmids were recovered and verified according their restriction patterns when digested using *Nde*l and *Hind*III. The restriction patterns were analyzed in agarose gel 0.8% (w/v) and then, the clones were sent for subsequent sequencing of the metagenomic inserts.

### *In silico* analysis of DNA inserts and identified protein sequences

Putative ORFs from the small fragment sequences were identified using ORF Finder program, available online in (http://www.ncbi.nlm.nih.gov/gorf/gorf.html). Comparisons between the insert amino acid sequences were performed against NCBI database using BLAST (https://blast.ncbi.nlm.nih.gov/Blast.cgi) alignment. There-dimensional models of the chimeric LacZ-α metagenomic peptides (NS1-NS9) and α -peptide LacZ were obtained from the ITASSER algorithm server (https://zhanglab.ccmb.med.umich.edu/I-TASSER/) (Zhang, 2008) and images were created with PyMOL (http://www.pymol.org/). Thermodynamic analysis of mRNA secondary structure from the different small DNA inserts was performed using the NUPACK algorithms (http://www.nupack.org/). The free energy of a given sequence in a given secondary structure was calculated using nearest-neighbor empirical parameters (Serra and Turner 1995; Mathews *et al.*, 1999; Zuker, 2003). For each construct, folding energy of an mRNA molecule was calculated from positions -4 to +70 nt relative to translation start of the *lacZ* gene, considering previous data (Kudla *et al.*, 2009) and positions of the DNA inserts (new DNA sequences started at position +53 nt). 

## Results

### Copy number of plasmids alters β-galactosidase expression and halo detection

Previously to the screening for enzymes in the selected SMA-PR media (Figure 1A), we carried out controls for testing the phenotype of clones carrying pSEVA232, the minimal and modular vector used in the construction of the metagenomic library (Figure 1B). For this, we streaked *E. coli* DH10B cultures carrying pSEVA232, pSEVA242, and pSEVA242-1.5 Kb insert within the MCS (multiple cloning site) on SMA-PR plates to obtain single colonies. After incubation of the plates for 24 h at 37 ºC we observed yellow halos around colonies just as in the clones carrying pSEVA242 (Table 1).

**Table 1:** Presence or absence of yellow halos indicative of vector-intrinsic β-galactosidase activity in bacterial clones bearing different plasmids.

Vector plasmid	Enzyme activity^a^	Copy number (copies per cell)	Origin of Replication
pSEVA242	Yes	High (100+)	pRO1600/ColE1
pSEVA242- 1.5 Kb insert	No	High (100+)	pRO1600/ColE1
pSEVA232	No	Medium (30-50)	pBBR1

a Visualization of a yellow halo around colonies after incubation in SMA-PR plates for 24 h at 37 ºC.

### Screening for proteases and glycosyl hydrolases in SMA-PR may lead to false positives

**Figure 2:**
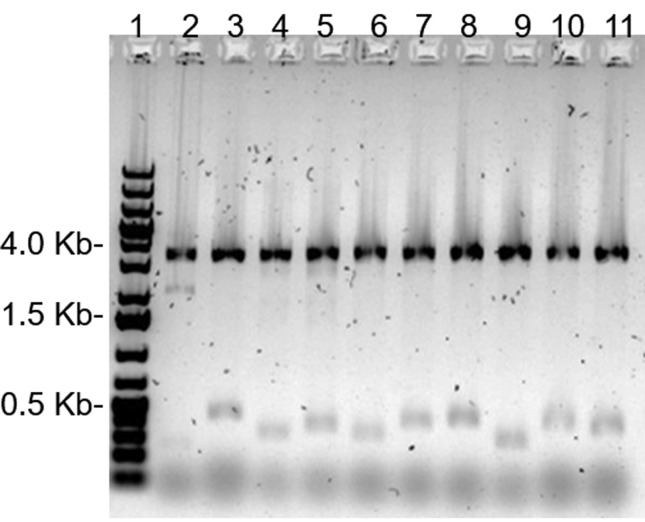
Restriction analysis of plasmids extracted from potential positive clones digested with HindIII and NdeI in agarose gel 0.8% (w/v). Line 1: molecular marker GeneRuler 1kb Plus DNA (Thermo Fisher – Waltham, EUA), line 2: empty pSEVA232; lines 3-11: plasmids extracted from clones NS1 to NS9.

In order to search for genes coding for proteases and GHs, we screened a metagenomic library hosted in *E. coli* DH10B, which was previously generated in our laboratory (Figure 1A). The screenings were carried out in SMA-PR media, supplemented with kanamycin 50 μg/mL, that allows to distinguish between GHs (yellow halos) and proteases (clear halos) activities (Figure 1A). From around 63,000 clones screened, we recovered 280 potential positives clones for GHs, of which, just nine maintained their phenotype when transferred to a new SMA-PR plate (i.e., colonies with yellow halos; Figure 1C). Re-transformed clones were tested for GH activity in SMA-PR plates and plasmids isolated from the colonies surrounded by yellow halos were digested with *Hind*III and *Nde*I enzymes, which revealed six recombinant plasmids with unique restriction patterns (Figure 2). Surprisingly, restriction profile analyses and sequencing of metagenomic inserts showed that the metagenomic fragments were too small (between 42 and 173 bp) for encoding enzymes able to display activity (Figure 2, Table 2, Table S1). It is important to highlight that the library was constructed using fragments of 2-7 kb and presented an average insert size of 4.08 kb, not showing plasmids with smaller fragments, when was initially tested for average insert size calculation.

**Table 2:** Metagenomic fragments contained in plasmid recovered from positive clones and their sequence features.

DNA fragment	Size (bp)	ORF length (aa)^a^	Truncated	Closest similar protein	Organism/ E-value^b^	ΔG (Kcal/mol)^c^	In-frame chimeric peptide^d^ (aa)
INS1	160	50	C-term	Unknown		-223.1 /-15.1	142
INS2	63	-				-166.5 /-16.6	102
INS3	148	18	C-term	Unknown		-212.7 /-17.1	110
INS6	116	37	N/C-term	DNA topoisomerase 4	*Pseudomonas aeruginosa* PAO1/ 3 E-07	-195.3 /-15.3	147
INS7	44	-				-165.3 /-14.2	123
INS9	125	38	N-term	Unknown		-201.7 /-18.0	150

a aa, amino acids.

b The sequences with an E-value of more than 0.001 in BLAST searches were considered to be unknown proteins.

c ΔG (Kcal/mol) of the mRNA secondary structure predicted by the NUPACK algorithms were calculated from the complete mRNA molecule (+1 of the transcription until the transcriptional terminator T_0_) or just considering a window spanning positions -4 to +70 nt relative to translation start. For the LacZα peptide values were -146.4 and -18.2 Kcal/mol, respectively.

d Chimeric peptide originated from the insertion in-frame of the metagenomic DNA into the *lacZ*α*-*gene, which originally encodes a 107aa peptide.

**Figure 3:**
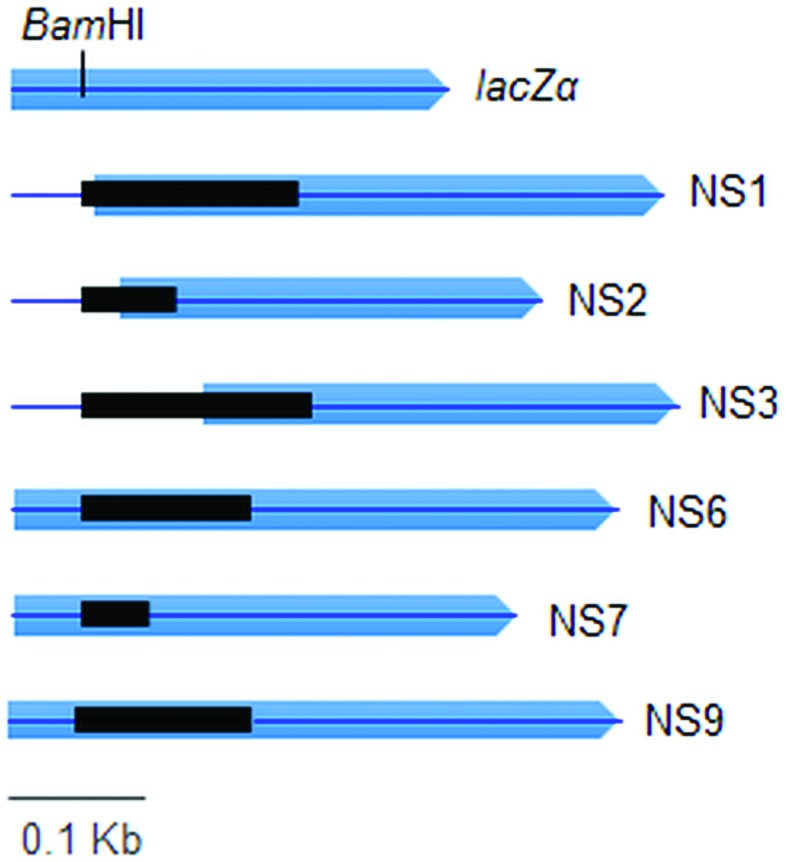
Chimeric LacZα/metagenomic peptides (NS1-NS9) resulted from the in frame metagenomic DNA insertion. In blue is shown the DNA sequence coding for the LacZa -peptide and in black the metagenomic insert, cloned in the BamHI restriction site. Complete (NS6, NS7 and NS9) and partial (NS1, NS2 and NS3) recovery of the LacZa -peptide were obtained after in frame DNA insertion.

**Figure 4:**
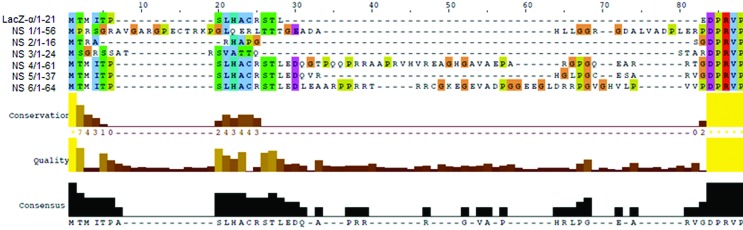
Alignment of the N-terminal region of the chimeric peptides (NS1-NS9) and LacZa-peptide. Alignment was carried out with the T-COFFEE Multiple Sequence Alignment Server and visualization was done with the Jalview program. In general, there were no amino acids conserved along the N-terminal sequence.

**Figure 5:**
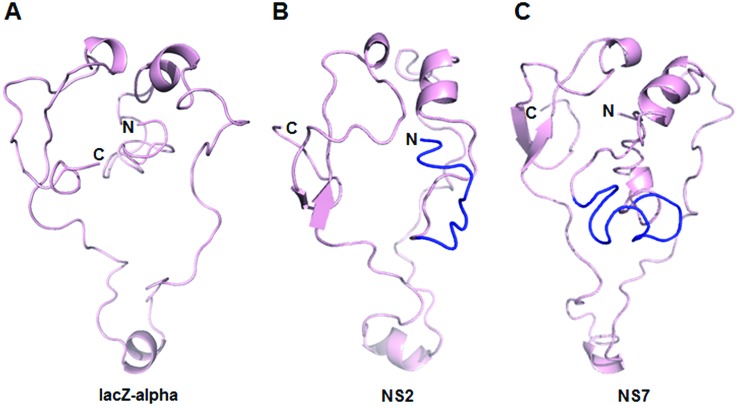
Structural models of the β-galactosidase LacZα-peptide (A) and chimeric peptides NS2 (B) and NS7 (C) resulted from the in frame metagenomic DNA insertion. In light pink is shown the 3D structure corresponding to the lacZa -peptide and in blue the metagenomic inserts. The ITASSER and PyMol softwares were used for structural model’s generation and visualization, respectively.

*In silico* analysis of the amino acid sequences (Figures 3 and 4) of the chimeric LacZα *-*fragment/metagenomic peptides resulted from the DNA insertion showed that DNA were inserted *in frame* within the coding region of the *lacZ*α -gene present in the original vector. Figure 3 shows that complete (DNA inserts NS6, NS7 and NS9) and partial (DNA inserts NS1, NS2 and NS3) recovery of the *LacZ*α-peptide were obtained after *in frame* DNA insertion. The N-terminal regions of the chimeric α -fragment/metagenomic peptides were aligned with the LacZα -peptide looking for conserved amino acids along the N-terminal sequence, although not a clear tendency was observed (Figure 4). On the other hand, three-dimensional modelling analysis of the chimeric peptides in comparison with the original LacZα -peptide provided initial evidence of an overall structure maintenance that should assure the activity of the chimeric α peptide when is added *in trans* (Figure 5, Figure S1). Taken together, these results indicated that the positives clones were the result of the recovery of functional lacZα -polypeptides, showing a strong limitation of the screening technique used.

### Reduced free energy in mRNA secondary structure could explain increased expression levels in metagenomic clones

**Figure 6:**
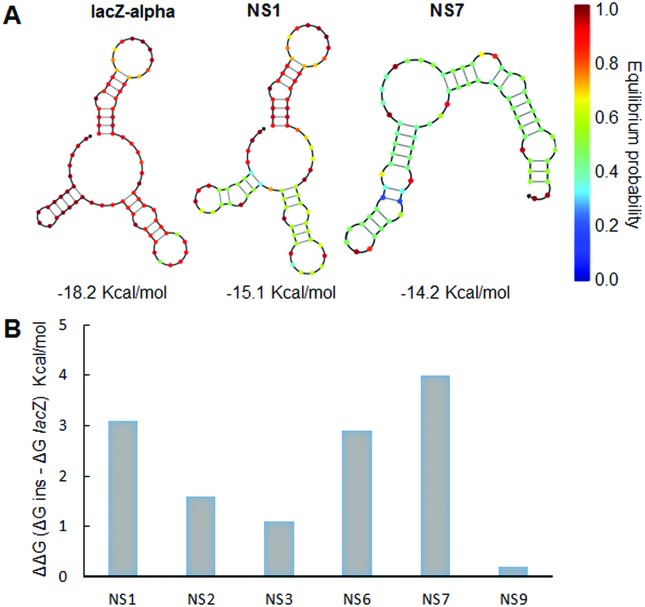
Recovery of clones with halos correlating to relative decreases in free energy of folding when a metagenomic fragment was inserted in the lacZ gene. A) Thermodynamic analysis at 37 ºC for a dilute solution containing the strand species that interact to form the possible ordered complexes (RNA secondary structures) using the NUPACK algorithms. For each construct, folding energy was calculated from positions -4 to +70 nt relative to translation start; three example structures are shown. B) Changes in free energy expressed as DDG considering DG values of predicted secondary structure of RNA with (DG ins) and without metagenomic inserts (DG lacZ) expressed in Kcal/mol.

In light of the evidence presented above, we hypothesized that the recovery of positive clones with very short DNA fragments should be an effect of the random generation of functional lacZα -fragments that are either more active than the original polypeptide or expressed at higher level. In order to comprehend the potential molecular mechanisms underlying the rise of false-positive clones, we combined literature information with the *in vivo* and *in silico* data obtained for the nine identified clones which were able to increase the expression of the *lacZ*α -gene contained in pSEVA232. Previous studies have shown that mRNA molecules less stable at the 5-end region are associated with a positive influence on protein expression (Kudla *et al.*, 2009; Gu *et al.*, 2010; Goodman *et al.*, 2013). To obtain evidence supporting the hypothesis that recovering of the nine positive clones was due to higher expression levels of the chimeric *lacZ*α *-*genes with respect to the original from pSEVA232 (with no phenotype in SMA-PR), we analyzed the local mRNA secondary structure of the different DNA inserts in comparison to the *lacZ*α -gene. Thus, for each construct (NS1-NS9 and *lac*Z without insert) we calculated the predicted minimum free energy (ΔG) associated with the secondary structure of its entire mRNA, or the 5-end region of its mRNA (Table 2). The folding energy of the entire mRNA did not show a reduction (Table 2). By contrast, the folding energy in position -4 to +70 nt relative to the translation start showed that in all the new sequences originated by metagenomic DNA insertion, the stability of the mRNA molecules was lower than the original, that is, with less negative ΔG values (Figure 6, Table 2).

## Discussion

In the present study, we used a metagenomic functional approach intending to recover two different types of enzymes in a single assay (i.e., GHs and proteases) using a methodology previously described in the literature (Jones *et al.*, 2007; Popovic *et al.*, 2015). For this, we used the vector pSEVA232 for library construction, since it displays unique features, such as being minimalist, synthetic, modular, and has a broad host-range (Silva-Rocha *et al.*, 2013). Plasmid pSEVA232 is a *lacZ*α*-*based plasmid, as most of the plasmids used in small-insert metagenomic libraries (Lämmle *et al.*, 2007; Mirete *et al.*, 2007; Guazzaroni *et al.*, 2013; Morgante *et al.*, 2015; Gao *et al.*, 2016; Zhou *et al.*, 2016). Prior to library construction, we check that plasmid pSEVA232 were not presenting β-galactosidase activity in SMA-plates. As shown in Table 1, we observed yellow halos around colonies just as in the clones carrying pSEVA242. These results were expected since pSEVA242 is a high copy number plasmid, carrying the β-galactosidase α -fragment in its backbone (Silva-Rocha *et al.*, 2013), which guarantees the proper expression of the LacZα -peptide and subsequent protein complementation. As the SMA-PR medium contains lactose, its hydrolysis by LacZ produces an acidic shift detected as a yellow halo (Figure 1A).

The molecular mechanism for blue/white screening (that is, recovering of functional β-galactosidase LacZ) is based on a genetic engineering of the *lac* operon in the *E. coli* chromosome (coding for the omega peptide with an N-terminal deletion) combined with a subunit complementation achieved with the cloning vector (coding for the α peptide) (Padmanabhan *et al.*, 2011). Thus, plasmid pSEVA242 encodes α peptide of LacZ protein, which bears an internal MCS, while the chromosome of the host strain (*E. coli* DH10B) encodes the remaining omega subunit to form a functional β-galactosidase enzyme upon complementation. On the other hand, the plasmid pSEVA242-1.5 Kb insert within the MCS of *lacZ*α *-*gene did not produce a yellow halo, as the α-fragment was disrupted. Finally, pSEVA232, although also being a *lacZ*α -based plasmid, carries a pBBR1 origin of replication, leading to a medium number of copies of plasmids per cell (Table 1), which does not allow enough expression of *lac*Z for proper phenotype production. This feature was essential for using the broad host-range pSEVA232 vector for library construction.

After the screening in SMA-PR we successfully obtained nine clones, among 63,000 screened clones, showing the typical yellow halos indicative of GH production. However, all of them were false positives, since small DNA fragments were inserted *in frame* within the *lacZ*α *-*gene present in the original vector (Figures 3 and 5). Here it is worth mentioning that the same metagenomic library was used for activity-driven screenings of β-glucosidases, which allowed the identification and biochemical characterization of a new enzyme (Alves L.F., Meleiro L.P., Silva R.N., Westmann C.A., Guazzaroni M.E., unpublished results). This data is important to show that the screen of the same library for other phenotypes allowed to properly recover clones for which it would be highly unlikely that short inserts into the lacZ gene would generate positive clones, meaning that this library is capable of yielding inserts with functional genes.

To understand the potential molecular mechanisms underlying the rise of false-positive clones, we analyzed the local mRNA secondary structure of the different DNA inserts in comparison to the lacZα -gene. Preceding studies have shown that the thermodynamic stability of mRNA secondary structure near the start codon can regulate translation efficiency in *E. coli* and other organisms, and that translation is more efficient the less stable the secondary structure (Kudla *et al.*, 2009; Gu *et al.*, 2010; Goodman *et al.*, 2013). Although codon bias has been related to slowing ribosomal elongation during initiation and lead to increased translational efficiency (Tuller *et al.*, 2010; Li *et al.*, 2012; Pechmann and Frydman, 2012), a recent systematic study using > 14,000 synthetic reporters in *E. coli* demonstrated that reduced stability in RNA structure, and not codon rarity itself is responsible for expression increases (Goodman *et al.*, 2013). In this sense, the molecular mechanistic explanation is that tightly folded mRNA obstructs translation initiation, thereby reducing protein synthesis (Kozak, 2005).

Our analyses showed that the stability of the mRNA molecules in all the new sequences originated by metagenomic DNA insertion was lower than the original, that is, presented more positive ΔG values, in position -4 to +70 nt relative to translation start (Figure 6, Table 2). Kudla *et al.* (2009) obtained similar results with respect to the region used for free energy calculation. In this context, studies showed that the region of strongest correlation between folding energy and expression did not overlap with the Shine-Dalgarno sequence (de Smit and van Duin, 1990; Kozak, 2005), but with the 30-nt ribosome binding site centered around the start codon (Kudla *et al.*, 2009). Therefore, results obtained here could explain the identification of the nine clones as positives in the screenings. Consequently, our data are in accordance with previous studies, which demonstrate that reduced mRNAs stability near the translation-initiation site had increased protein expression (Kudla *et al.*, 2009; Gu *et al.*, 2010; Goodman *et al.*, 2013).

## Supplementary material

The following online material is available for this article:

Table S1 - Sequences of the metagenomic DNA inserts derived from the extracted plasmids of potential positive clones

Figure S1 - Structural models of the chimeric peptides.
