# Supplementary material for: Boundaries in metagenomic screenings using lacZα-based vectors
Source: Genet Mol Biol. 2020 Mar 6;43(1):e20180252. doi: 10.1590/1678-4685-GMB-2018-0252 (PMC7198016; doi:10.1590/1678-4685-GMB-2018-0252)
Supplement: Supplementary file 1 [file 1415-4757-GMB-43-1-e20180252-s1.pdf]

Supplementary Material to "Boundaries in metagenomic screenings using *lac*Z*α*-based vectors"

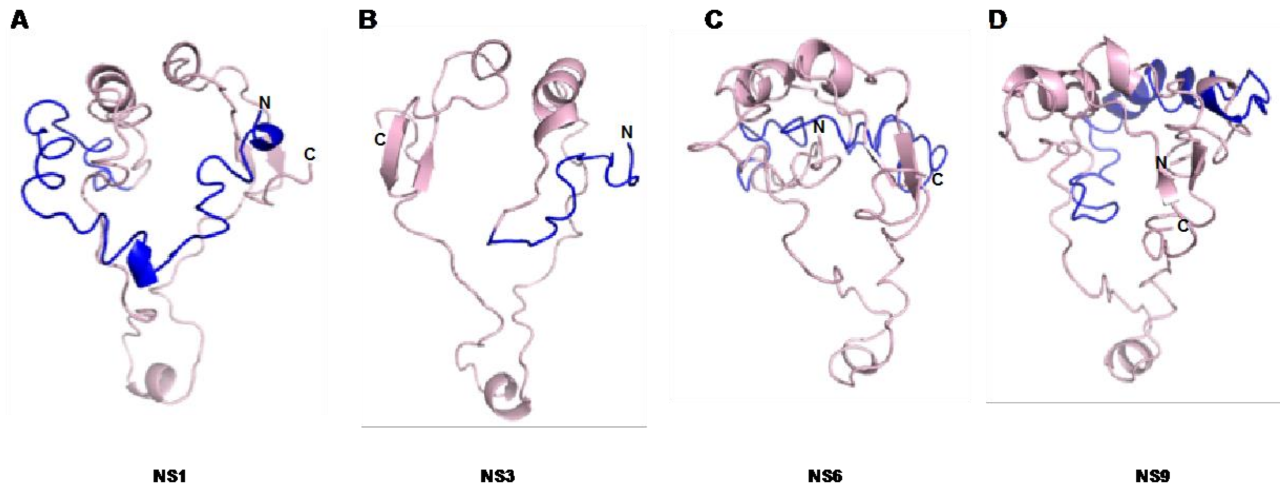

Figure S1 – Structural models of the chimeric peptides NS1 (A), NS3 (B), NS6 (C) and NS9 (D) resulted from the in frame metagenomic DNA insertion. In light pink is showed the 3D structure corresponding to the lacZα peptide and in blue the metagenomic inserts. The ITASSER and PyMol softwares were used for structural model's generation and visualization, respectively
