# Supplementary material for: Boundaries in metagenomic screenings using lacZα-based vectors
Source: Genet Mol Biol. 2020 Mar 6;43(1):e20180252. doi: 10.1590/1678-4685-GMB-2018-0252 (PMC7198016; doi:10.1590/1678-4685-GMB-2018-0252)
Supplement: Supplementary file 2 [file 1415-4757-GMB-43-1-e20180252-s2.pdf]

Supplementary Material to "Boundaries in metagenomic screenings using *lac*Z*α*-based vectors"

Table S1 - Sequences of the metagenomic DNA inserts derived from the extracted plasmids of potential positive clones. Derivative amino acid sequences of the predicted *in frame* translated peptides are also shown. Amino acids from the metagenomic inserts are in bold.

Insert	DNA metagenomic sequence	Predicted *in frame* peptide (aa)
NS1 (160 bp)	GTGCCGTATGCCGAGGTCAGGTCGAGCGGTCGGAGCTCGAGGACCCGAGTGCACCCGCAAGCCCGGCCTGCAGGAACGACTGACGACCACCGGCGAAGCGGATGCCCATCTTCTCGGCGGTCGTGGCGACGCGCTCGTTGCCGACCCGCTGGAGCGCCCG	**MPRSGRAVGARGPECTRKPGLQERLTTTGEADAHLLGGRGDALVADPLERP**DPRVPSSNSLAVVLQRRDWENPGVTQLNRLAAHPPFASWRNSEEARTDRPSQQLRSLNGEWRLMRYFLLTHLCGISHRIWCTLSTICSDAA
NS2 (63 bp)	CTCATCGGCGCCATCAACTTCGTGAACCTCATGACACGCGCGCGGCACGCGCCGGGCAGTGGA	**MTRARHAPGSG**DPRVPSSNSLAVVLQRRDWENPGVTQLNRLAAHPPFASWRNSEEARTDRPSQQLRSLNGEWRLMRYFLLTHLCGISHRIWCTLSTICSDAA
NS3 (148 bp)	CGGCGCCAGTTGCACGACCTTCTCCCACGCCGTCAAGGTTCCGACGCCGACGACGGCGCCGGATACCGCGGTCCGCCCAGCGCCTGGAGATATGTCCGGTCGGTCGTCGGCAACTCGTTCTGTCGCGACCACGCAATCGACAGCCCGC	**MSGRSSATRSVATTQSTAR**DPRVPSSNSLAVVLQRRDWENPGVTQLNRLAAHPPFASWRNSEEARTDRPSQQLRSLNGEWRLMRYFLLTHLCGISHRIWCTLSTICSDAA
NS6 (116 bp)	AAGGAACGCCTCAACAGCCGCGACGCGCTGCGCCTCGTGTCCACGTACGTGAAGCCGGCCATGGAGCTGTGGCTGAACCAGCACGTGGACCTGGGCAGGAAGCTCGCCGAACTGGT	MTMITPSLHACRSTLED**QGTPQQPRRAAPRVHVREAGHGAVAEPARGPGQEARRTG**DPRVPSSNSLAVVLQRRDWENPGVTQLNRLAAHPPFASWRNSEEARTDRPSQQLRSLNGEWRLMRYFLLTHLCGISHRIWCTLSTICSDAA
NS7 (44 bp)	AGGTGCGTCACGGGCTTCCCGGGTGCGAGAGTGCGCGCGTGGGC	MTMITPSLHACRSTLED**QVRHGLPGCESARVG**DPRVPSSNSLAVVLQRRDWENPGVTQLNRLAAHPPFASWRNSEEARTDRPSQQLRSLNGEWRLMRYFLLTHLCGISHRIWCTLSTICSDAA
NS9 (125 bp)	TCGAAGCTGCGCGGCCGCCCAGGCGAACACGAAGATGCGGAAAAGAAGGCGAGGTCGCCGACCCTGGTGGTGAGGAAGGCCTTGACCGCCGCCCGGGCGTTGGCCACGTCCTCCCAGTAGTGCCC	MTMITPSLHACRSTLED**LEAARPPRRTRRCGKEGEVADPGGEEGLDRRPGVGHVLPVVP**DPRVPSSNSLAVVLQRRDWENPGVTQLNRLAAHPPFASWRNSEEARTDRPSQQLRSLNGEWRLMRYFLLTHLCGISHRIWCTLSTICSDAA
